# EurocanPlatform, an FP7 project of the European Commission—first year commentary

**DOI:** 10.3332/ecancer.2012.ed13

**Published:** 2012-03-13

**Authors:** Gordon McVie, Ulrik Ringborg

**Affiliations:** 1 European Institute of Oncology, Milan, Italy; 2 The Karolinska Institutet, Solna, Sweden

EurocanPlatform is an exciting collaboration between Europe’s top cancer centres to accelerate the pace of taking new scientific results to the patients’ bedside. This is being achieved by building shared platforms between the partners in each critical area of cancer research from causation of cancer to delivery of novel therapeutic strategies in clinical trials.

The first year of EurocanPlatform has been remarkably free of glitches. Most work packages are up and running well, inevitably a couple are getting up to speed now. The first general assembly at the end of the first year was exciting and rich in content. More than a dozen talks outlined work in hand, and outlined future plans. The most stimulating aspect was the obvious willingness of scientists from the various member institutes (23 in all) to open their technological cupboards, and in the true spirit of the project share know-how and facilities with colleagues.

Several innovative hypothesis driven trials were presented by scientists, not clinicians - probably the most remarkable memory of the meeting. And the spread of the platforms from aetiology of cancer through prevention to clinical cost efficacy was abundantly evident.

On the former topics, cancer risk assessment and prevention, two projects were discussed and supported. The first was exciting as it harnessed new technological areas, epigenetics and metabolomics to populations, normal, or at risk. IARC in Lyon is looking for epigenetic markers of early cancer using large cohorts, while the EPIC study, the largest global prospective study of diet and other lifestyle factors will be exploited to hunt patterns in metabolomics, which might also prove to be useful early signals of cancer or premalignancy. The EPIC study has recruited over 520,000 volunteers and over 2000 incident cases of lung cancer have already been noted. The intention is to take biological specimens (plasma/urine) and use high resolution mass spectrometry to unravel the molecular patterns in low and high consumers of foods of interest. In parallel, the food metabolome will be assessed with particular attention to those substances, and interactions between these and other biometric parameters will be tested for utility as future biomarkers.

Also in the area of biomarkers, Oslo (OCCC) and Milan (INT), covered extensively the molecular profiling of normal, premalignant and cancerous tissue samples from lung, ovary and pancreas. Preliminary findings on 419 glycan-related genes have been presented. The same team leads an ambitious project to refine the technology of identifying and exploiting circulating tumour cells (CTCs). The early application of this technique has shown that numbers of tumour cells retrieved from blood samples correlate with tumour bulk, and changes in numbers have mirrored response to therapy, and subsequent regrowth. Finding small numbers of circulating cells in early disease such as breast cancer has so far been disappointing, but second generation technology is being used by several groups in the consortium and better sensitivity and selectivity are confidently predicted. If one regards CTCs as easily accessible “biopsies” of tumours it is immediately apparent how useful they might be to provide information of metastatic potential, resistance to therapy, and eventual sensitivity to new targeted molecules. First however the group is planning to retrieve gene signatures from CTCs to check whether they are identical or not to the parent cancer. Gene profiling has been achieved so far on as few as 50 CTCs, so this approach looks promising.

The translational nature of the EurocanPlatform was confirmed by description of two hypotheses driven clinical trials mooted by Amsterdam (NKI), Paris (Curie), Paris (IGR), Cambridge (CRUK), Barcelona (Val d’Hebron) and London (ICR), each based on collaborative laboratory projects amongst the partners. They include an early phase trial of a dual PI3K/mTOR inhibitor together with tamoxifen to reverse hormone resistance in metastatic breast cancer patients whose cancers are HER2 negative and Estrogen receptor positive. Then there is a plan for a test of the new Braf inhibitor Vemurafenib, shown to be effective in melanoma patients whose tumours display a mutation in BRAF V600E, but in colorectal patients instead of melanoma. It seems that bowel tumours treated with an inhibitor of this mutated gene switch on EGFR which is the target for a number of agents including cetuximab, so a combination of the two agents is logical to trial.

The sharing of platform technology know-how, and open access to partner institute facilities is the very essence of EurocanPlatform. A number of collaborations have already been initiated thanks to the project, including kinome resequencing (particularly in ovarian cancer) and refinement of RNAi technology led by Amsterdam (NKI), SNP genotyping led by Cambridge (CRUK), and Reverse Phase Protein Arrays (RPPA), led by Paris (Curie).

The interlinking of clinicians and scientists has clearly been achieved already in the project, the sharing of technologies amongst scientists is evident, and the cross talk between clinical epidemiologists expert in prevention, clinicians and scientists, especially focussed on biomarker development bodes well for the future positive impact on cancer patient outcomes. The outreach and dissemination of the results of the project are the responsibility of an ongoing Education programme led by Bari (IT), and the public and professional communications are the responsibility of *e*cancer.org.

The following organisations are also involved in EurocanPlatform: the European CanCer Organisation (ECCO), European Organisation for Research and Treatment of Cancer (EORTC), the European Cancer Patient Coalition (ECPC) and the Organisation of European Cancer Institutes (OECI).

